# The OmpA of commensal *Escherichia coli* of CRC patients affects apoptosis of the HCT116 colon cancer cell line

**DOI:** 10.1186/s12866-022-02540-y

**Published:** 2022-05-19

**Authors:** Mahsa Mirzarazi, Soroor Bashiri, Ali Hashemi, Mahmoud Vahidi, Bahram Kazemi, Mojgan Bandehpour

**Affiliations:** 1 Student Research Committee, Department of Medical Biotechnology, School of Advanced Technologies in Medicine, Shahid Beheshti University of Medical Sciences, Tehran, Iran; 2 Department of Pathology, School of Medicine, Shahid Beheshti University of Medical Sciences, Tehran, Iran; 3 Department of Microbiology, School of Medicine, Shahid Beheshti University of Medical Sciences, Tehran, Iran; 4 Department of Medical Laboratory Sciences, School of Allied Medical Sciences, Aja University of Medical Sciences, Tehran, Iran; 5 Cellular and Molecular Biology Research Center, Shahid Beheshti University of Medical Sciences, Tehran, Iran

**Keywords:** Colorectal cancer, Microbiota, *Escherichia coli*, B2 phylogenetic group, Outer membrane protein A, Apoptosis

## Abstract

**Background:**

Colorectal cancer ranks third globally among all types of cancers. Dysbiosis of the gut microbiota of people with CRC is one of the effective agents in the tumorigenesis and metastasis in this type of cancer. The population of *Escherichia coli* strains, a component of gut microbiota, is increased in the gut of people with CRC compared with healthy people. So, *E.coli* strains isolated from these patients may have a role in tumorigenesis. Because the most isolated strains belong to the B2 phylogenuetic group, there seems to be a linkage between the bacterium components and malignancy.

**Material and methods:**

In this study, the proteomic comparison between isolated *Ecoli* from CRC patients and healthy people was assayed. The isolated spot was studied by Two-dimensional gel electrophoresis (2DE) and Liquid chromatography-mass spectrometry (LC–MS). The results showed that the expression of Outer membrane protein A (OmpA) protein increased in the commensal *E.coli* B2 phylogenetic group isolated from CRC patients. Additionally, we analyzed the effect of the OmpA protein on the expression of the four genes related to apoptosis in the HCT116 colon cancer cell line.

**Results:**

This study identified that OmpA protein was overexpressed in the commensal *E.coli* B2 phylogenetic group isolated from CRC patients compared to the *E.coli* from the control group. This protein significantly decreased the expression of *Bax* and *Bak,* pro-apoptotic genes, as well as the expression of *P53* in the HCT116 Cell Line, *P* < 0.0001. LC–MS and protein bioinformatics results confirmed that this protein is outer membrane protein A, which can bind to nucleic acid and some of the organelle proteins on the eukaryotic cell surface.

**Conclusions:**

According to our *invitro* and *insilico* investigations, OmpA of gut *E.coli* strains that belong to the B2 phylogenetic group can affect the eukaryotic cell cycle.

## Background

Colorectal cancer, or CRC, is the third most common cancer globally, affecting 1.36 million people each year [1–3]. CRC has a weak prognosis [4], and only a small number of people are diagnosed in the early stages of this cancer. Therefore, an early diagnosis for proper treatment and malignancy prevention are essential [5]. Many factors, such as genetics, lifestyle, and environment, play an important role in increasing the incidence of CRC [6–8]. It has been proven that a change in the diversity of the gut microbiota population or the digestive dysbiosis affects the process of the emergence and progression of malignancy in intestinal cells [1, 2, 9]. The human body works in symbiosis with microbiota [3, 5].

Studies have found that the *Escherichia coli* (*E.coli*) load increased in the intestines of people with colon cancer, and the pathogenic mechanisms and type of toxins production differ from the intestinal isolates in healthy people [9, 10]. This bacterium is divided into four major phylogenetic groups, A, B1, B2, and D. Most isolates with more severe virulence and pathogenicity factors are in groups B2 and D, respectively [10, 11]. Most of the B2 phylogenetic group strains can prevent apoptosis pathway progression in colon cells. Consequently, it can play a role in the high proliferation of these cells and cancer advancement [12, 13]. Therefore, group B2 isolates may be considered a real risk factor for the development of colorectal cancer. In research on the mucus and biopsy specimens from people with colon cancer and diverticulosis, scientists found that most of these isolates, especially those containing the target genotoxins, belonged to the B2 phylogenetic group. Also, they concluded that these toxins could play a role in the process of tumorigenesis [14]. According to other documents, EPEC diarrhea-causing *E.coli* isolates can secrete a protein called ESPF. This protein inhibits the factors involved in the repair of DNA mismatch mutations [15]. Similar studies performed on the role of the structural and secretory proteins of the bacterial isolates in tumor formation have reached similar results to the above studies [13, 16].

To achieve a hypothetical microbial biomarker for CRC screening, we compared and analyzed the proteomics of *E.coli* isolated from two groups of people, one with CRC and the on from the healthy population; the results gave us a microbial protein biomarker called OmpA. Furthermore, given the importance of the role of this bacterium on the apoptosis pathway of the colon cancer cell line, we investigated the effect of this protein on the HCT116 cell line to better understand how to change the expression level of the pathway’s essential *Bak*, *Bax*, *Bcl-2*, and *P53* genes.

## Results

### Characteristics of samples

Fecal samples (stool) were collected from 20 people (9 females and 11 males) who found colorectal cancer in their biopsy during a colonoscopy but before any treatment. More sampling was necessary to use statistical analysis to compare isolated *E.coli* proteins from people who have colorectal cancer with the proteome of *E. coli* isolated from healthy people. Thus, stool samples of 50 (25 females and 25 males) healthy people were collected. It should be noted that only the *E.coli* strains were isolated from each sample [17].

### The *uspA* gene amplification

Using *uspA* gene fragment amplification and sequencing (LC639828) confirmed that all 70 isolates of bacteria from the control and patient groups were *E.coli*.

### Identification of *E. coli* phylogenetic groups

We identified 20 isolates of gut microbiota *E.coli* in the CRC patients, and 11 (55%), 7 (35%), 0 (0%), and 2 (10%) of these strains belonged to the B2, D, B1, and A phylogenetic groups, respectively. Of the 50 commensal *E. coli* isolates from the healthy group, 13 (26%), 12 (24%), 5 (10), and 20 (40%) bacteria were allocated into the B2, D, B1, and A groups, respectively. It is worth noting that the prevalence of *E.coli* in the B2 phylogenetic group was higher in people with CRC (*P*v = 0.02). Moreover, in both the control and patients groups, 3 (6%) and 6 (30%) of *E.coli* strains in the B2 phylogenetic group were isolated from people who had family members with cancer, and this difference was statistically significant, *P*v < 0.05.

### Two-dimensional gel electrophoresis

**Fig. 1:**
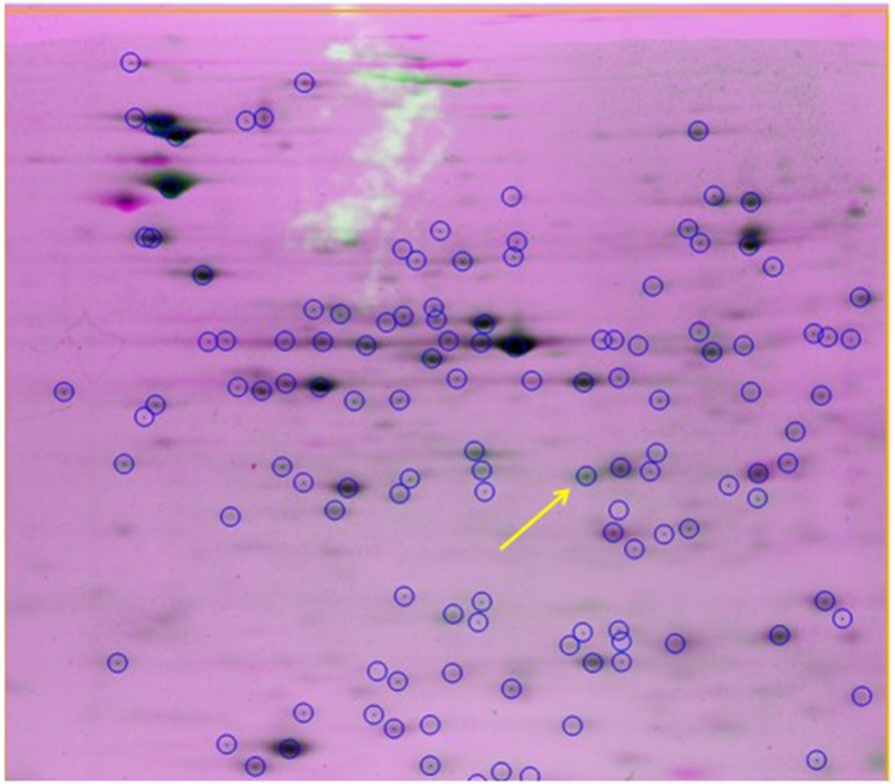
The results of the 2DE gel analyzed by Same Spot software. CRC group gel spots (black) were merged with control group gel spots. The marked spot indicates that green is predominant over pink, and the software quantitatively confirmed this result

**Fig. 2:**
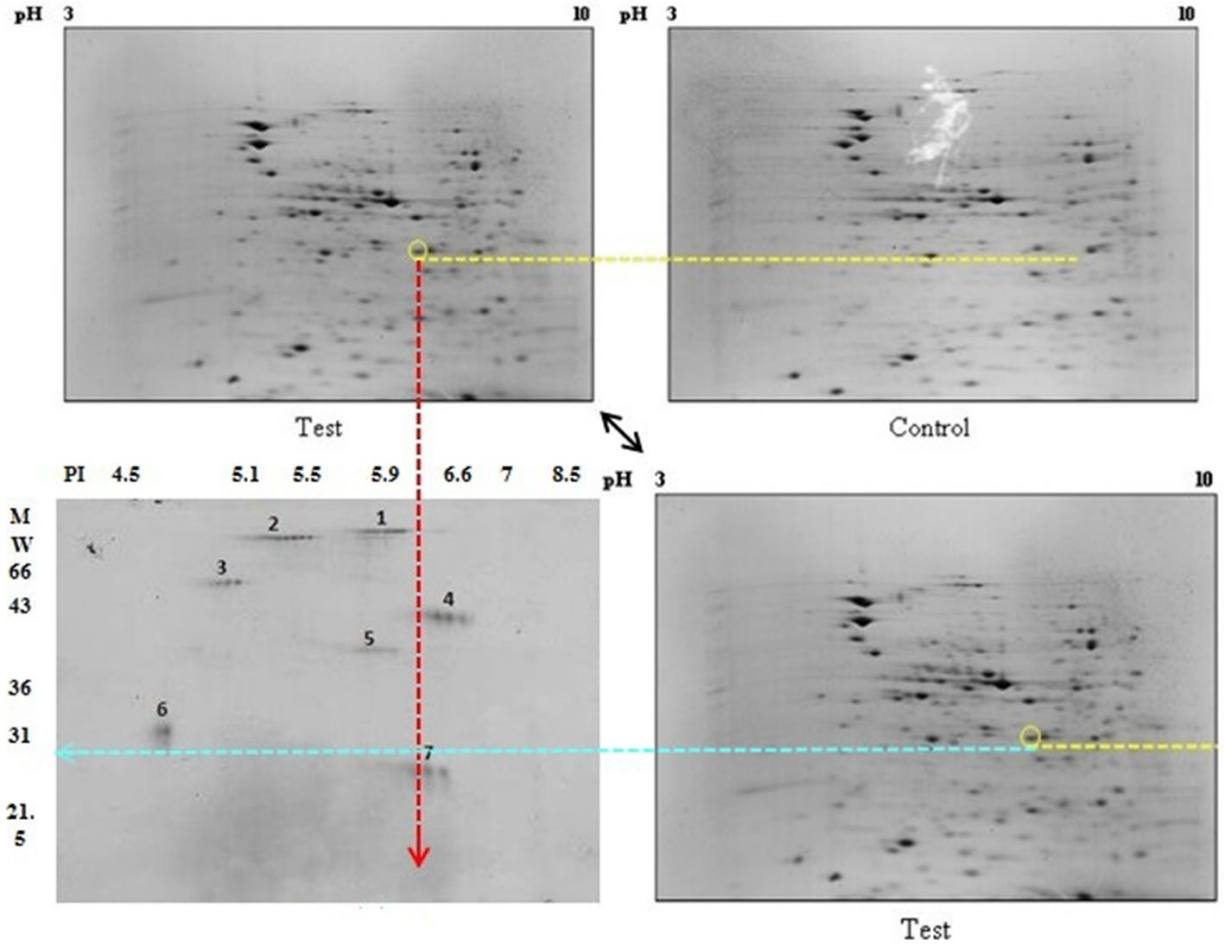
The results of 2DE electrophoresis of *E.coli* B2 phylogenetic bacterium isolated from control and CRC groups. **a** 2DE gel belongs to *E.coli* proteins isolated from the CRC group, (**b**) 2DE gel belongs to *E.coli* proteins isolated from the control group, and (**c**) Protein size Marker. The yellow dashed line shows the difference between the two gels. Also, the chosen protein was labeled. The red dashed line shows the PI approximately, and the blue dashed line shows the approximate weight

Using the Same Spot software, we analyzed the spot results in the 2DE gels on the pooled samples from the control and CRC groups qualitatively and quantitatively. By merging the 2DE gel images from both groups, the software recognized a distinct spot. This spot in the CRC 2DE gel was much darker and denser than the same spot in the other group (Fig. 1). The figure revealed a weight of about 30 kD and PI ~ 6 for the chosen protein (Fig. 2).

### LC–MS outputs analysis

Based on results analysis from the Mascot server (the standard for protein identification using mass spectrometry data), five proteins were found with high sequence homology to the target protein. The datasets used to align the sequences were obtained from the NCBI (www.ncbi.nlm.nih), UniProt, and Expasy (www.expasy.org) web servers. Among them, three proteins with higher scores and the highest similarity rate were most similar to the target protein (Table 1).

**Table 1:** The list of proteins that were most similar to the target protein based on the Mascot server outputs

*The rank of protein and Mascot code*	*Name*	*Organism*	*Score*	*Queries matched*	*PI*	*mass*
sp|A0A2S4N3N0|OMPA_SHIFL	Outer membrane protein A(OmpA	*Shigella flexneri*	517	15	5.31	*37,260*
sp|P02935|OMPA_SHIDY	Outer membrane protein A(OmpA)	*Shigella dysenteriae*	497	13	5.31	*37,718*
sp|Q0TCN0|MDH_ECOL5	Malate dehydrogenase	*E.coli* O6:K15:H31 (strain 536 /UPEC)	391	10	5.61	*37,260*

### Target protein identification by Bioinformatics analysis

After aligning the protein sequences, results showed the similarity between the malate dehydrogenase strain 536 of UPEC to the strain K12 of *E.coli* protein was 100%. However, the similarity of mitochondrial mdh in human cells (mdh2) with the mdh of the *E.coli* strain was only 99%. Malate dehydrogenase is an essential enzyme in eukaryotes and prokaryotes, i.e., *E.coli*. mdh is one of the tricarboxylic acid (TCA) cycle enzymes that protects bacteria against oxidative stress and reactive oxygen species (ROS) [18].

In all cancers, such as CRC, the ROS increases in the epithelial cells [19]. Subsequently, it has been seen that *E.coli,* as a symbiosis microenvironment around these cells in the gut microbiota, produces malate dehydrogenase. This enzyme has a 99% similarity to mdh2. The mitochondrial mdh or mdh2 in the cancer cells' ROS decreases the expression of the genes involved in apoptosis escape and metastasis [20–22]. Following this line of thought, because mdh inhibits cancer cells, the target protein in this study is not malate dehydrogenase; moreover, its Mascot similarity score was lower than other proteins (Table 1).

It is noteworthy that in addition to the high Mascot similarity scores (Table 1), the sequence similarity of the outer membrane protein A of *E.coli* K12 strain and the same protein from *Shigella flexneri* and *Shigella dysenteriae* was 93% and 85%, respectively. OmpA is a protein found in the membrane of most bacteria, such as *Enterobacteriaceae*, which facilitates the bacterium's adherence to the epithelial cell and has a role in protecting and ensuring the bacteria’s survival [23, 24].

### Insilico analysis of the OmpA protein

**Fig. 3:**
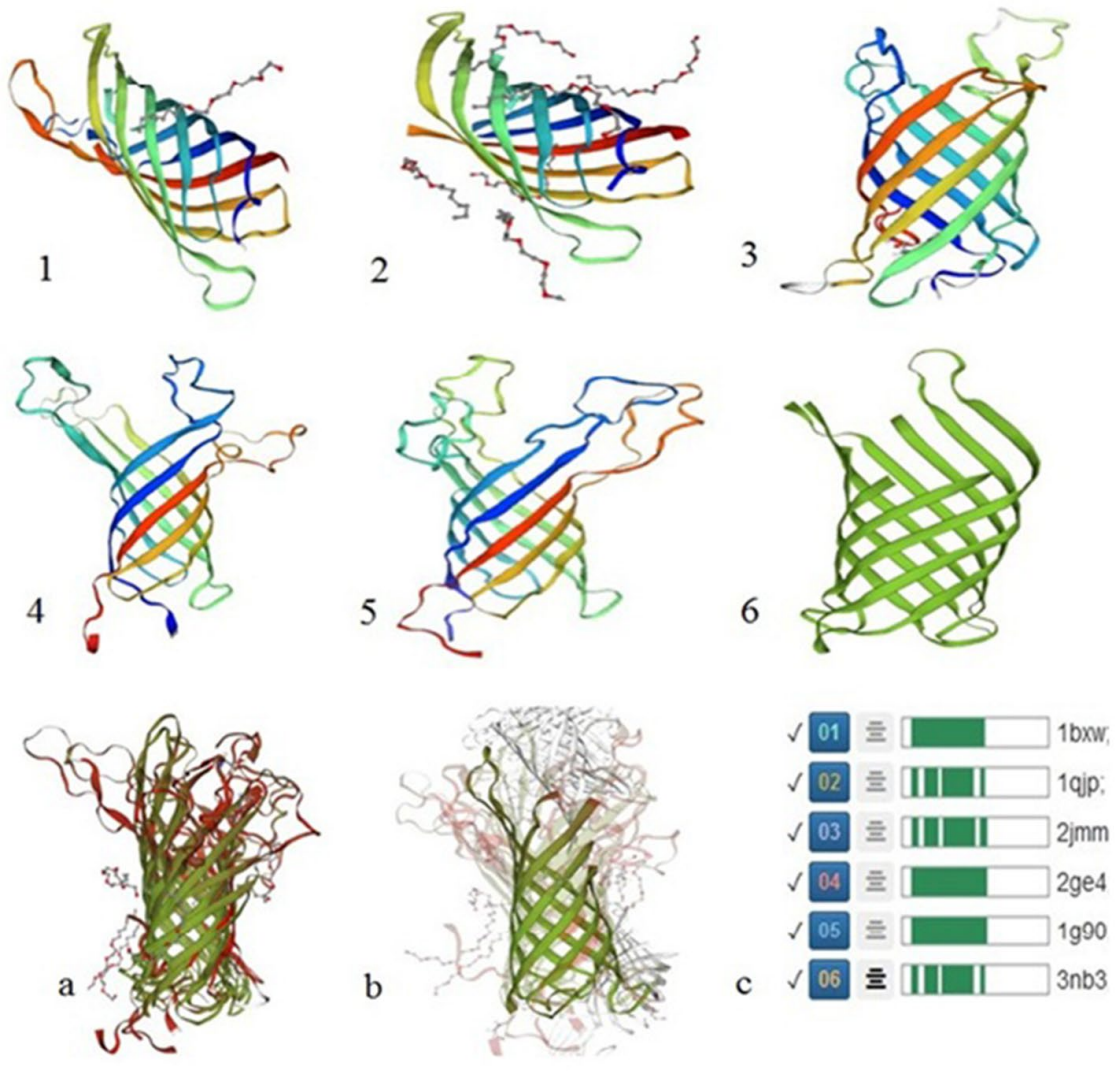
The comparison of 3D structures of the OmpA in *E.coli* K12 strain and *Shigella flexneri*. 1 (1bxw), 2 (1qjp), 3 (2jmm), 4 (2ge4), 5 (1g90), and 6 (3nb3). **a** The merge and comparison of 3D structures of the OmpA in *E.coli* K12 strain by the SWISS-MODEL server. **b** The merge and comparison of the OmpA 3D structure of *Shigella flexneri* with five 3D structures of the OmpA of *E.coli* K12 strain. **c** The similarity of 3D structures of OmpA from the SWISS-MODEL Repository

Figure 3 shows the conformational structures of the OmpA protein belong to the *E.coli* K12 strain. The 2ge4 [25], 1g90 [26], 2jmm [27], 1qip [28] and 1bxw [29] PDB files were obtained from the PDB databank. The 3nb3 PDB file or OmpA protein structure of *Shigella flexneri*was obtained [30]. The similarity of these proteins in both strains was shown by merging and comparing their 3D structures in the SWISS-MODEL homology-modeling server (Fig. 3).

The overview results of organic enzyme cofactors from the COFACTOR database confirmed the molecular function (MF) of the Omp A asporin activity (GO:0,015,288), with a 0.99 score. Furthermore, he biology process (BP) of this protein predicted by gene ontology (GO) showed that OmpA is a transporter (c score:1.00) and has a role in the cellular process (c score:0.90) and response to stimulus and stress (c score: 0.80, and 0.79). The protein’s role in stress response explains its overexpression in bacteria around cancer cells microenvironment compared to bacteria around healthy intestinal epithelial cells.

**Fig. 4:**
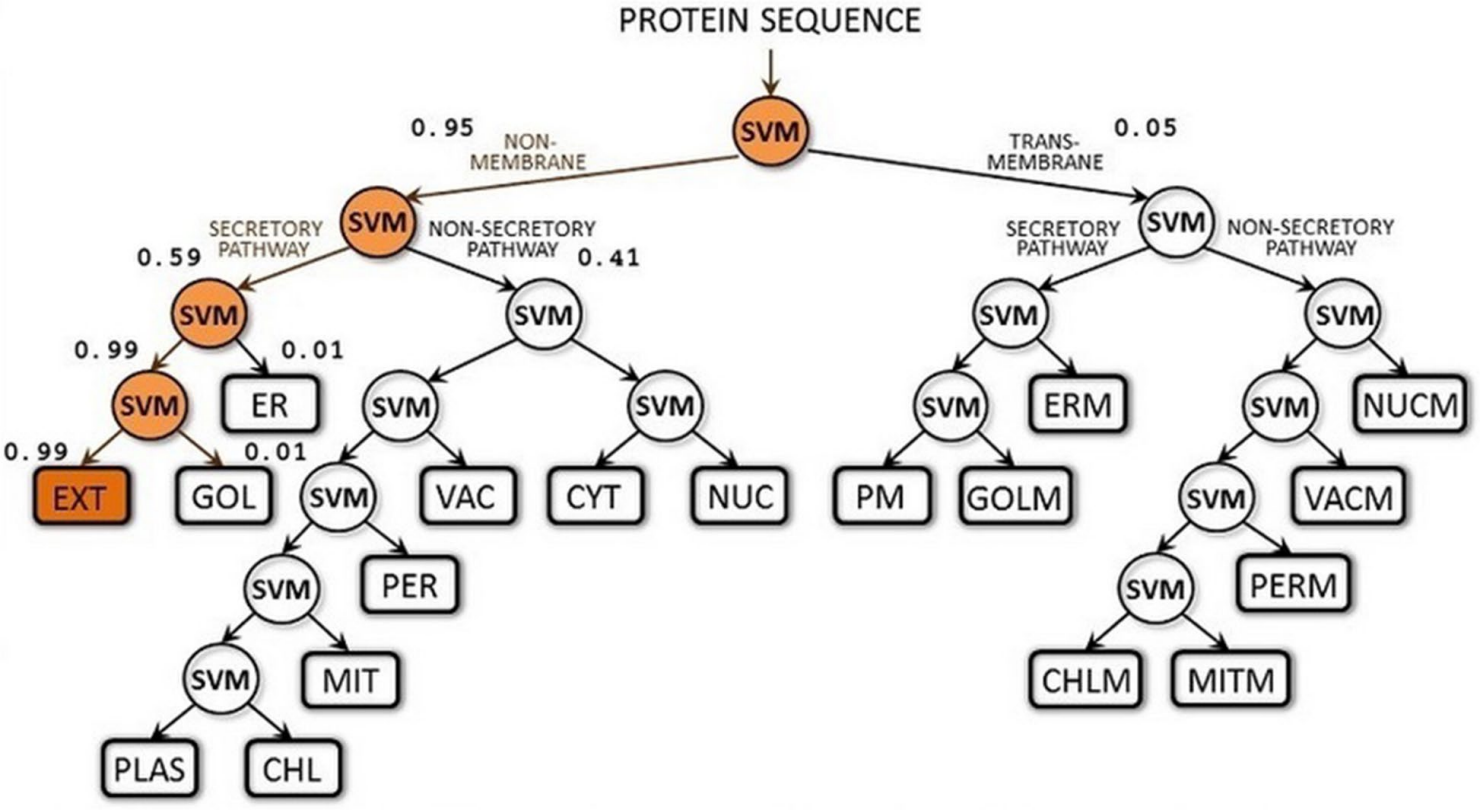
The diagram of the LocTree3 analysis of OmpA target proteins in the eukaryotic cells. SVM: support vector machine, EXT: extra-cellular, ER: endoplasmic reticulum, GOL: golgi apparatus, VAC: vacuole, PER: peroxisomes, MIT: mitochondria, CHL: chloroplast, CYT: cytosol, NUC: nucleus, PM: plasma membrane, GOLM: golgi apparatus membrane, ERM: endoplasmic reticulum membrane, NUCM: nucleus membrane, VACM: vacuole membrane, PERM: peroxisomes membrane, MITM: mitochondria membrane, CHLM: chloroplast membrane

The diagram of the LocTree3 analysis (see Fig. 4) illustrates that the OmpA is an extracellular protein (score: 0.99) that can target the proteins of cytoplasm, nucleus, peroxisomes, and mitochondria (score:0.41).

**Fig. 5:**
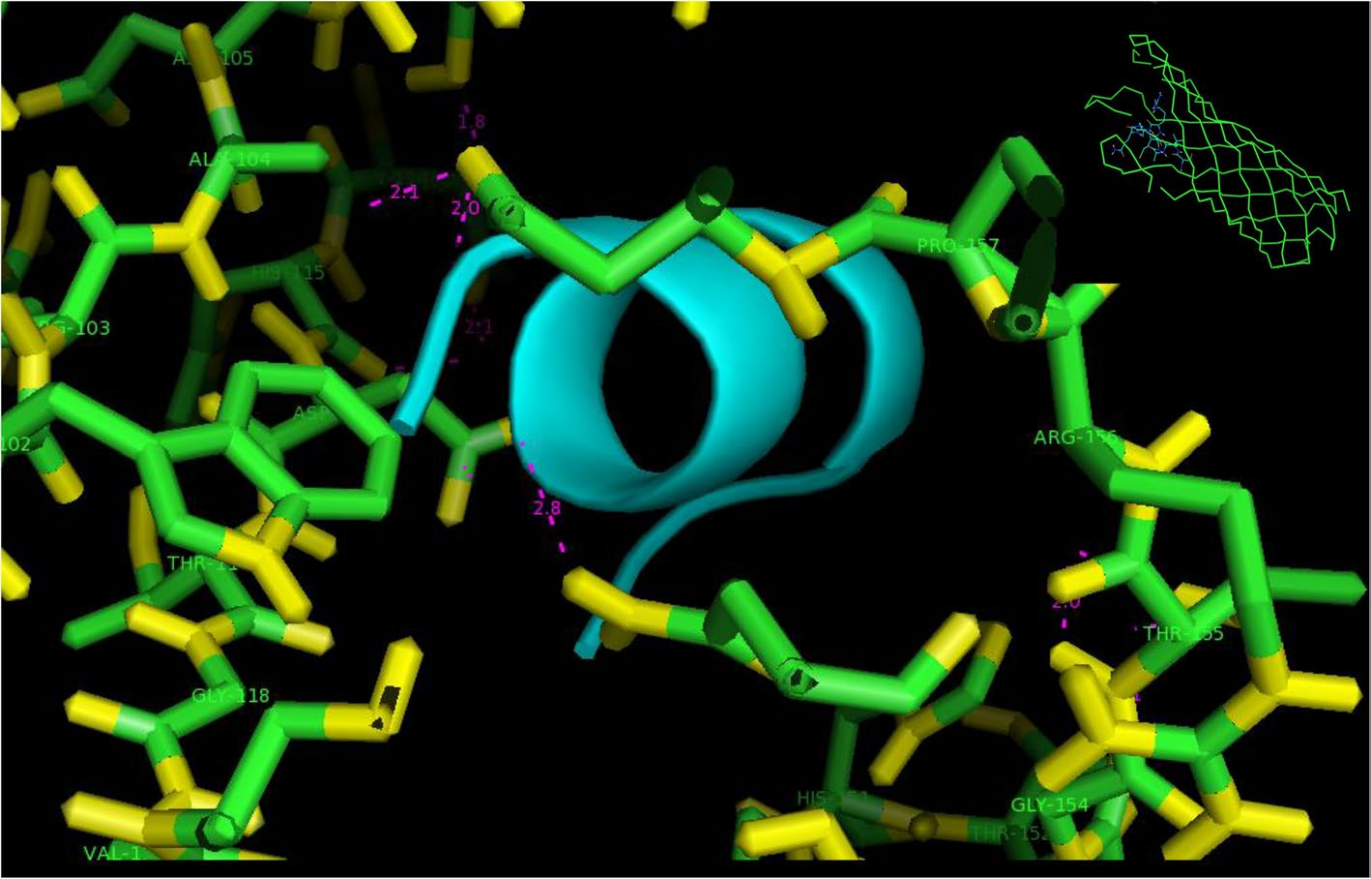
The 3D model predicted by the COACH server for the state of the OmpA binding to a peptide

The predicted structure matched the 4erh PDB code using the protein–ligand binding site COACH server [31]. The match was with the crystal structure of the OmpA domain from *Salmonella enterica* subsp (Fig. 5). According to this modeling, binding sites of the residues of OmpA for binding to the peptide occurred at 165, 166, 201, 202, 204, 205, 206, 210, 213, 217, 264, and 268 positions, and binding sites of the OmpA for binding to the nucleic acid were at 202, 203, 245, 247, 248, 250, 251, 255, 260, 264, and 267 residues. While using the Predict Protein database, the residues in binding sites of OmpA protein to DNA and RNA were at positions 33–100 and 33–66, respectively.

### Treatment of the HCT116 Cell Line with the OmpA protein

**Fig. 6:**
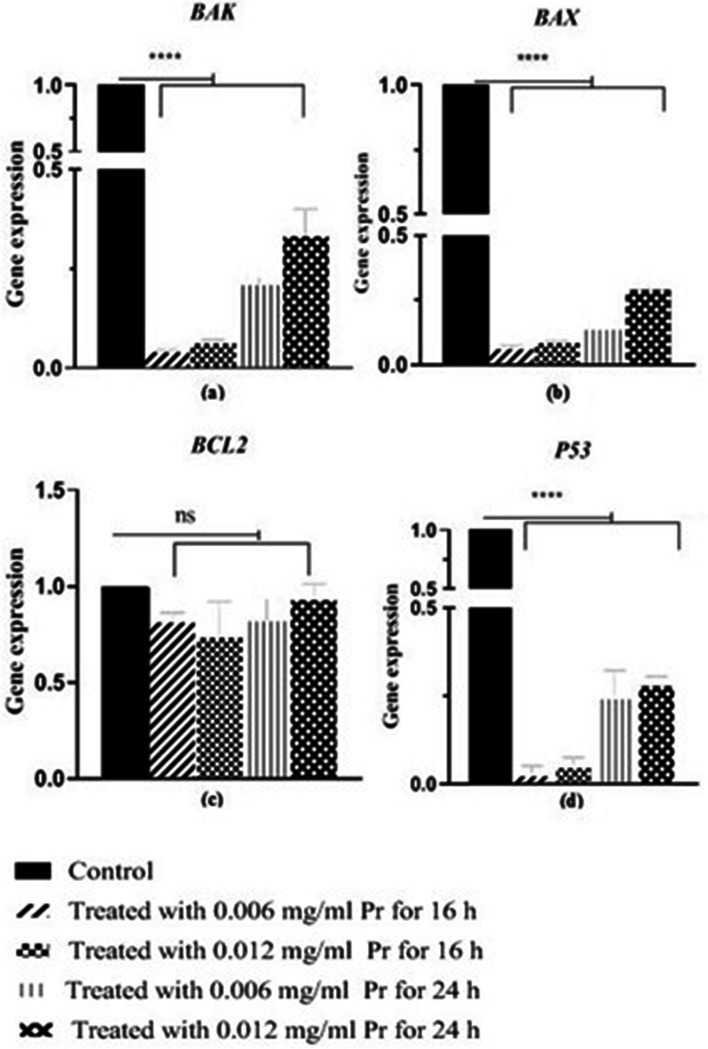
Real-time PCR analysis of *BAK* (**a**), *Bax* (b), *Bcl-2* (**c**), and *P53* (**d**) genes expression in groups of the HCT116 cell line by normalizing against the corresponding levels of β-actin,**** shows significant differences of the level of gene expression in the treatment groups compared to the control group, with *P* < 0.0001. ns means the absence of significant differences in the gene expression level in the treatment groups compared to the control group *P* > 0.05

**Fig. 7:**
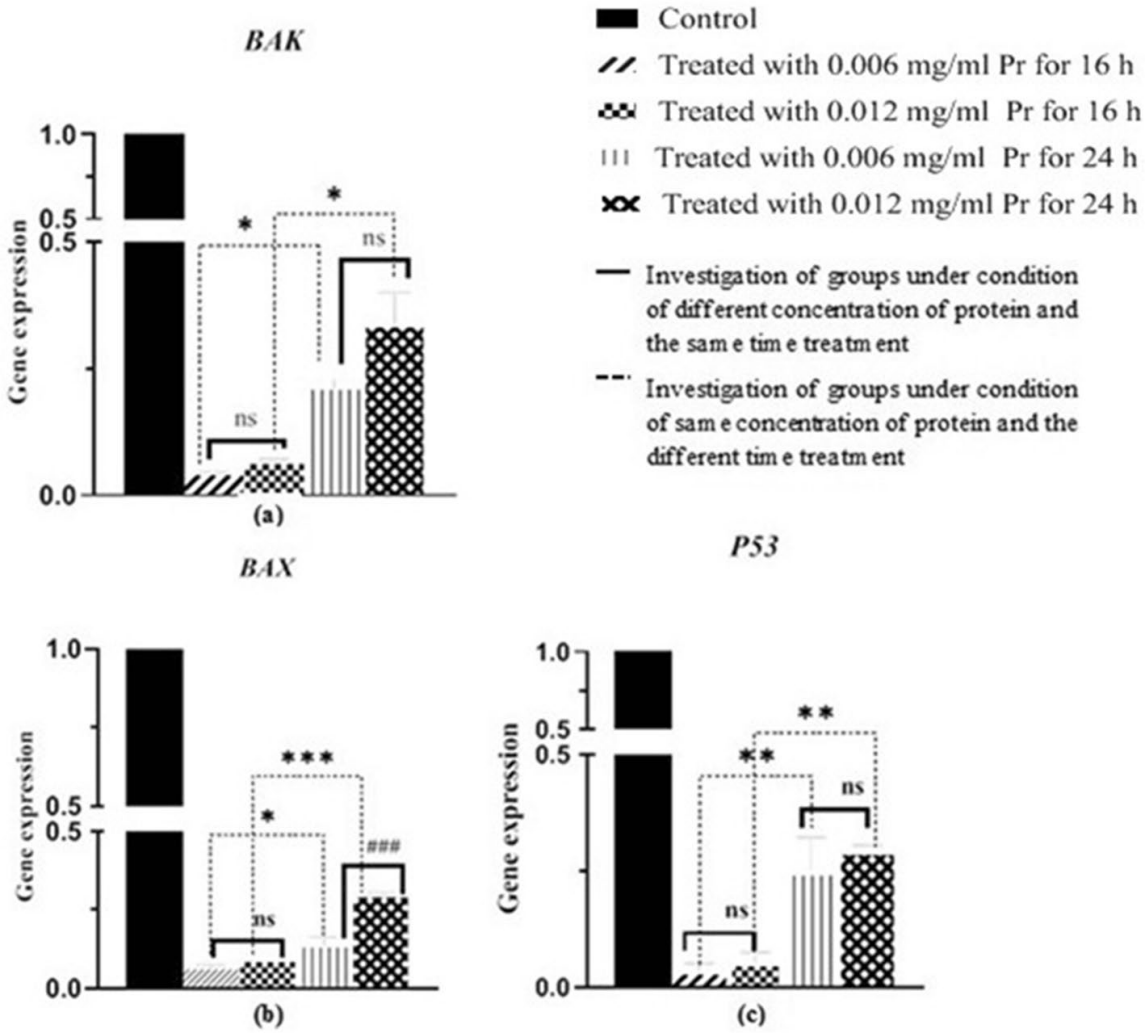
Real-time PCR analysis of *BAK* (**a**), *Bax* (**b**), and *P53* (**c**) gene expression based on time treatment and protein concentration. **a** * = difference of the level of *BAK* expression between the treated groups in a condition of equal protein (0.12 µg) concentration and different times was 0.015, and * = difference of the level of gene expression between the treated groups in a condition of equal protein (0.25 µg) amount and different times was 0.002. ns = no significant differences in genes expression level based on the condition of different protein concentrations and the same treatment time *P* > 0.05. **b** * = differences of the level of *BAX* expression between the treated groups in a condition of equal protein (0.12 µg) concentrations and different times was 0.029, and *** = differences of level of genes expression between the treated groups in a condition of equal protein (0.25 µg) amount and different times was 0.0002. ### = differences in the level of genes expression based on the condition of different protein concentrations and the same treatment time (24 h) was 0.0009. **c** ** = differences of the level of *P53* expression between the treated groups in a condition of equal protein (0.12 µg) concentrations and different times was 0.0084, and ** = differences of the level of gene expression between the treated groups in a condition of equal protein (0.25 µg) concentrations and different times was 0.0067

The effects of the OmpA protein concentration and treatment time on the expression of *Bak*, *Bax*, *Bcl-2*, and *P53* genes in the HCT116 cell line were investigated using real-time PCR analysis. Results showed that treating the HCT116 cell line with OmpA protein led to a significant down-regulation in the expression of *Bak*, *Bax*, and *P53* genes, *P* < 0.0001. However, a significant difference was not seen in the expression of *Bcl-2* in the four treated groups compared to the control group, *P* > 0.05 (see 6). As can be seen in Fig. 7, the treatment time had a more significant effect on the Bak, Bax, and *P53* genes expression than the protein concentration. In Fig. 6, the analysis showed a statistically significant difference in the expression level between the genes Bak, Bax, and p53 and between the control and the HCT116 cell line groups treated with OmpA (Fig. 6). The difference is significant in both groups for the HCT116 cell line treated for 16 and 24 h. The results presented in Fig. 7 indicate that the 16-h treatment of HCT116 cell lines with ompA had more effect on down-regulating the expression of these genes (Fig. 7).

## Discussion

CRC ranked third among cancers and correlates with the gut microbiota population [1, 32]. The dysbiosis of gut microbiota is an effective agent for tumorigenesis and metastasis in people with this type of cancer [1, 2, 9]. Recently, it was found that *E.coli* strains isolated from the gut microbiota of CRC patients play a role in tumorigenesis [33, 34]. Then again, most of these strains belong to the B2 phylogenetic group [11, 14], which has more severe virulence factors [35, 36]. DNA damage and oncogenes activation have been proven to trigger the intrinsic apoptotic pathway. In this situation, the tumor suppressor *P53* is induced and subsequently activates pro-apoptotic genes, *Bak* and *Bax.* Apoptosis depends on the *Bax* and *Bcl-2* genes expression balance. However, the *Bcl-2,* as an antiapoptotic gene, overexpresses in solid tumors and increases the cells' proliferation [37]. The overexpression of the *Bax* or underexpression of the *Bcl-2* can indicate cell apoptosis [38].

The role of these genes is effective in the apoptotic pathway. So, we assayed the effect of a protein of *E.coli* on their expression. We analyzed the proteome of the isolated *E.coli* strains from CRC patients’ stool using 2DE [39, 40]. The results identified the outer membrane protein A, or OmpA protein, was significantly overexpressed in the CRC group isolated *E.coli*. This protein was characterized using the LC–MS, a valid, sensitive, and careful method to identify bacterial proteins [40–42]. As shown in Fig. 3, the OmpA of *Shigella flexneri* has a 93% similarity to OmpA of *E.coli* strains. The insilico study showed that OmpA has an overall beta-barrel structure composed of an anti-parallel beta-strand. Short turns connect these strands on the membrane's periplasmic part and extended loops on the extracellular part [43].

On the one hand, based on the GO terms, this protein handles the response to stress conditions for the bacterium (GO: 0,050,896 and GO: 0,006,950); and the other hand, in pathogen *E.coli* strains, this protein is considered a virulence factor [23, 36, 44]. This proves that OmpA can stimulate the immune system [24]. It is worth noting that those *E.coli* strains that belonged to the B2 phylogenetic group have the most virulence factors [35, 45, 46]. So it is reasonable to conclude that because of the ROS stress conditions in these strains, which have a symbiosis with cancer cells, OmpA overexpresses and emerges as a virulence factor. The LocTree3 analysis illustrated that in addition to the extracellular proteins, some eukaryotic cell organelles' proteins, especially in the nucleus and mitochondria, are targeted by OmpA (score: 0.41). One bioinformatic study on proteins of pathogenic *E.coli* strain by LocTree3 analysis showed many targeting proteins in eukaryotic organelles for these strains [47]. They found that with time any alteration in the folding and function of these proteins or DNA damage could affect the cycle of eukaryotic cells infected by pathogenic *E.coli* strains. This, in turn, can drive the cells to proliferation and cancer development [15, 47].

This protein causes a significant down-regulation of the expression of Bax, Bak, and p53 genes, *p* < 0.0001. According to the results mentioned in this study and the role of *BCL-2* in the apoptosis pathway, it is logical to state that this protein does not interfere with the downregulation of the *BCL-2* gene, *p* > 0.05.

## Conclusion

This study found that the overexpression of OmpA in the *E.coli* strains belonging to the B2 phylogenetic group isolated from the gut microbiota of the patients with CRC can affect the colon cell apoptosis pathway and drive the metastasis phenomenon.

## Materials and methods

### Samples preparation and culture

Fecal samples collected before any treatment from 20 people referred to Shahid Beheshti University of Medical Sciences affiliated medical centers after being diagnosed with colorectal cancer during a colonoscopy by the pathologis to isolate *E.coli* strains. Each sample was cultured on Mac Conkey agar and Eosin Methylene Blue agar plates (Merck, Germany) using the standard loop and incubated for 24 h at 37˚C. Statistical analysis was conducted to compare the isolates of *E.coli* of people with colorectal cancer and the *E.coli* isolated from healthy people. Thus, similar to the CDC patients, stools were collected, cultured, and *E.coli* strains were isolated from 50 people without any symptoms or relevance to the disease [17].

### DNA extraction and Molecular confirmation of *E.coli* strains

We first extracted the genomic DNA using the phenol–chloroform protocol [48] to identify the cultivated bacteria on Mac Conkey agar and EMB agar plates. Then, we amplified the uspA gene (universal stress protein-coding) as an *E.coli-*specific gene by the PCR method [49, 50] in the reaction conditions presented in Table 2. and Finally, we categorized the bacteria by sanger sequencing.

**Table 2:** Nucleotide sequence of primers and temperature conditions

**Genes**	**Size(bp)**	**Primers (5'- 3'**)	**Annealing Temp**	**Reference**
***uspA***	884	F: CGATACGCTGCCAATCAGT	60 C˚	[51]
		R: CGCAGACCGTAGGCCAGAT		
***chuA***	279	F: ACGAACCAACGGTCAGGAT	58 C˚	[36]
		R: GCCGCCAGTACCAAAGACA		
***YjaA***	211	F:TGAAGTGTCAGGAGACGCTG	55 C˚	
		R:ATGGAGAATGCGTTCCTCAC		
***Tsp*E4C2**	152	F: AGTAATGTCGGGGCATTCA	57 C˚	
		R:CGCGCCAACAAAGTATTACG		

### Identification of the isolated *E.coli* strains phylogenetic groups

The phylogenetic groups of all isolate *E.coli* bacteria were investigated with PCR performed for *chuA*(Outer membrane hemin receptor ChuA), *YjaA*(stress response protein)*,* and *Tsp*E4C2(Tail-specific protease)genes (Table 2) [35, 51, 52]. In addition, the EcoR62 strain of *E.coli* containing the chuA, yjaA, and TspE4C2 genes, as the positive control, was considered in this study [17, 51].

### Protein preparation and SDS–polyacrylamide gel electrophoresis (SDS-PAGE)

Before performing 2DE, we preferred to compare the two groups’ SDS-PAGE pattern of separated proteins in the isolated *E.coli*. So, after culturing the identified bacterial samples in nutrient broth, the B2 strains were pooled in each group and separated from the culture medium by centrifugation. Then, the bacterial samples were suspended in a lysis buffer (glycerol 10%, Tris (pH 8), PMSF 10 mM, and 1% Triton X-100) (Merck, Germany). After sonication of bacteria, total protein was precipitated using acetone (Merck, Germany). Next,the protein separation was performed by centrifugation (9056 g). Finally, total protein was incubated with protein loading buffer (Tris, Glycerol, SDS. Bromophenol blue, and DTT) (Merck, Germany) at 85c˚ 5 min [53]. A 12% (v/v) SDS-PAGE was used for all samples, and the gel was eventually stained with Coomassie brilliant blue R-250 [54, 55].

### Protein preparation and two-dimensional gel electrophoresis (2DE)

To prepare the samples, equal volumes of the 24-h culture in nutrient broth medium (Merck, Germany) of identified *E.coli* samples in the B2 phylogenetic group of both control (13 isolates) and CRC (11 isolates) groups were pooled separately. The bacterial sediments separated from the culture media were suspended in lysis buffer (Tris–HCl, EDTA, urea, 10% glycerol, DTT, and NP4O). The *E.coli* extracted protein samples concentration was 2.6 µg/mL for both the control and CRC group. The sample volume used in this section for both groups was 10 µL. Seven-centimeter immobilized pH gradient (IPG) strips with a nonlinear range of pH 3–10 (BioRad, USA) were used for isoelectric focusing (IEF). The rehydration step of the IPG strips was overnight. The rehydration buffer volume (7 M urea, 2 M thiourea, 4% CHAPS, 0.2% Bio-Lyte pH 3–10, 50 mM DTT, and a trace amount of bromophenol blue) for both the control and test group was 115 μL. The Protein IEF cell (BioRad) was focused by a linear increase from 0 to 250 V for 20 min, followed by a linear rise to14000 V. Afterward, the IPG strips were equilibrated by a 3 ml buffer (50 mM Tris–HCl pH 8.8, 6 M urea, 20% glycerol, 2% SDS, 0.01% bromophenol blue, and 2% DTT) for 15 min. Then, 3 ml of equilibration buffer devoid of DTT supplemented with 2.5% iodoacetamide was used to alkylate the samples (15 min). For the second dimension of electrophoresis, equilibrated strips were placed on top of the 12% SDS-PAGE gel, and electrophoresis was run in the following conditions ( 90 min and100 V). Finally, the gels were stained with Coomassie brilliant blue R-250 [56, 57]. The 2DE gel analysis and the decision to opt for a target protein were assayed by the Same Spot software version 5.1.012(U.K).

### Extraction of protein spot from the 2DE gel

First, the target protein spot in the 2DE gel was carefully cut, then crushed with an applicator, and sterile deionized water was added to dissolve the protein. Next, the protein solution was incubated for 1 h at 37° C. Then, the solution was passed through a ClearSpin filter microtube with a porosity of 0.22 µm(CLEARLINE Co., US) and centrifuged at 16,099 g for 3 min. Lastly, the supernatant containing the target protein was separated. The protein solution was sterilized using a 0.45 µm filter [58], and the protein concentration was measured with a nanodrop spectrophotometer (0.60 mg/ml).

### Protein identification by liquid chromatography-mass spectrometry (LC–MS)

In the first stage, the alkylation of the protein sample was carried out using DTT; in other words, it was digested by trypsin, and then according to standard protocols, peptides were extracted. The process utilized an automated liquid sampler, quaternary pump, degasser, column compartment, and a diode array detector (observed at 220 nm) controlled by the ChemStation© software (Agilent Technologies) on a Hewlett Packard Series1100 HPLC. Finally, advanced mass spectrometry was performed using a 4800 MALDI TOF/TOFTM analyzer (Applied Biosystems/MDS SCIEX) [59, 60]. Result analysis was performed using the Mascot server http://www.matrixscience.com/.

### Bioinformatics studies on LC–MS analysis results

The results of peptide sequences by the Mascot server in Table 2 were aligned using UniProt (www.UniProt.org/ and blast P (www.ncbi.nlm.nih/) to confirm and identify the target protein. The FASTA format of the protein sequences of bacteria were used for the alignments and comparison.

### Bioinformatics studies on *E.coli* target protein

Based on the NMR assay or X-ray crystallography, the 3D structure of OmpA was defined in the PDB format available in the UniProt database. Therefore, the protein's three-dimensional structure was evaluated in the *E.coli* K12 strain and *Shigella flexneri* by aligning the primary structure of the proteins using the SWISS-MODEL server (https://swissmodel.expasy.org/repository/uniprot/). Also, we decided to use the COFACTOR server (https://zhanglab.ccmb.med.umich.edu/COFACTOR2/ ) to find the molecular function (MF) and biological process (BP) of *E.coli* ompA, which are the parts of the gene ontology (GO).

We used an advanced support vector machine (SVM) and the LocTree3 server (https://rostlab.org/services/loctree3/ ) to predict the localization of the OmpA protein binding sites in eukaryotic cell organelles [47]. Using the Predict Protein Database (https://predictprotein.org/), we identified the OmpA surface residues that can bind to DNA and RNA. The COACH server (https://zhanglab.ccmb.med.umich.edu/COACH/) predicted two 3D models of the OmpA in the state of binding to a peptide and nucleic acid and determined the binding position of each of the residues of OmpA. COACH is a meta-server approach to protein–ligand binding site prediction. Starting from the given structure of target proteins, COACH will generate complementary ligand binding site predictions using two comparative methods, TM-SITE and S-SITE, which recognize the ligand-binding templates BioLiP protein function database by binding-specific substructure and sequence profile comparisons.

### Cell culture

We considered the human colorectal cancer HCT116 cell line (ATCC) as an invitro model to investigate the bacterial protein's effect. One hundred five cells were seeded inside a 6-well plate and cultured in an RPMI 1640 medium containing 10% fetal bovine serum (FBS) and 1% penicillin–streptomycin at 37 °C with 5% CO2 [58, 61].

### Treatment of HCT116 colon cancer cell line with the target protein

We categorized five groups of cell lines for the present study. Group one was treated with 0.006 mg/mL (10µL) target protein and incubated for 16 h. Group two was treated with 0.012 mg/mL (20µL) target protein and incubated for 16 h. Group three was treated with 0.006 mg/mL(10µL) target protein and incubated for 24 h. Group four was treated with 0.012 mg/mL (20µL) target protein and incubated for 24 h. The last group was the control group without any treatment.

Before any treatment with the identified protein, RPMI complete mediums were replaced with a FBS-free RPMI culture medium.

### RNA extraction, cDNA preparation, and genes expression analysis by Real-time RT-PCR

The RNA of the cell lines in each group was extracted using a kit (All Gene ® -Hybrid-RTM, Korea), and the cDNA templates were synthesized from 1 µg RNA with the oligo dT primer and reverse transcriptase enzyme (Fermentas, USA). Four apoptosis gene expression levels for the treated and control HCT116 cell line (Table 3) were analyzed. The Real-time PCR reaction mixture consisted of a 12 µl total volume solution containing 6 µl SYBR Premix EX Taq (2X) Master Mix and 0.25 µl ROX (50X) (Takara, Japan), 1 µl (10 µmol) forward and reverse primers (Metabion- Germany), 4 ng cDNA template, and PCR-grade H2O up to 12 µl. The reaction was conducted on a StepOne plus PCR system (Applied Biosystems, Foster City, CA). The conditions for the PCR test were 95 °C for 10 min, followed by 40 cycles of 95 °C for 10 s, 56 °C for 15 s, and 72 °C for 25 s. Primer specificity was verified by melting curve analysis. It should be noted that β-actin was considered the internal reference gene. The comparative quantification Ct method (∆∆Ct) evaluated the mRNA expression levels [38].

**Table 3:** The expression of Apoptosis genes evaluated in this study

Gene	Primer sequence (5ʹ-3ʹ)	Product length (bp)
***βActin***	F: GTTGGAGTGGATCCGCCGCACAA	224
	R: ATCCTGCCCTCAGAGGGCATGAA	
***Bax***	F: CGGGGAGCAGCCCAGAG	112
	R: CCCAGTTGAAGTTGCCGTCAG	
***Bak***	F: CAACCGACGCTATGACTCAG	152
	R: AGCCGAAGCCCAGAAGAG	
***Bcl-2***	F: GCGACTCCTGATTCATTGG	162
	R: GTCTACTTCCTCTGTGATGTTG	
***P53***	F: AGCACTAAGCGAGCACTG	156
	R: CTGGGCATCCTTGAGTTCC	

## Data Availability

http://getentry.ddbj.nig.ac.jp/getentry/na/LC639828/?format=flatfile&filetype=html&trace=true&show_suppressed=false&limit=10

## References

[1] Yu J, Feng Q, Wong SH, Zhang D, Liang Qy, Qin Y (2017). Metagenomic analysis of faecal microbiome as a tool towards targeted non-invasive biomarkers for colorectal cancer. Gut.

[2] Han S, Gao J, Zhou Q, Liu S, Wen C, Yang X (2018). Role of intestinal flora in colorectal cancer from the metabolite perspective: a systematic review. Cancer Manag Res.

[3] Lucas C, Barnich N, Nguyen HTT (2017). Microbiota, Inflammation and Colorectal Cancer. Int J Mol Sci.

[4] Mizoguchi E, Iyadorai T, Mariappan V, Vellasamy KM, Wanyiri JW, Roslani AC (2020). Prevalence and association of pks+ E.coli with colorectal cancer in patients at the University Malaya Medical Centre, Malaysia. PloS One.

[5] Villeger R, Lopes A, Veziant J, Gagniere J, Barnich N, Billard E (2018). Microbial markers in colorectal cancer detection and/or prognosis. WJG.

[6] Murphy N, Moreno V, Hughes DJ, Vodicka L, Vodicka P, Aglago EK (2019). Lifestyle and dietary environmental factors in colorectal cancer susceptibility. Mol Aspects Med.

[7] Almeida CVD, Camargo MRd, Russo E, Amedei A (2018). Role of diet and gut microbiota on colorectal cancer immunomodulation. WJG.

[8] O’Keefe SJ. Diet, microorganisms and their metabolites, and colon cancer. Nat Rev Gastroenterol Hepatol. 2016;13(12):691–706. 10.1038/nrgastro.2016.165 PMC6312102 27848961

[9] Bonnet M, Buc E, Sauvanet P, Darcha C, Dubois D, Pereira B (2014). Colonization of the human gut by E. coli and colorectal cancer risk. Clin Cancer Res.

[10] Wassenaar TME (2018). coli and colorectal cancer: a complex relationship that deserves a critical mindset. Crit Rev Microbiol.

[11] Veziant J, Gagniere J, Jouberton E, Bonnin V, Sauvanet P, Pezet D (2016). Association of colorectal cancer with pathogenic Escherichia coli: focus on mechanisms using optical imaging. World J Clin Oncol.

[12] Shimpoh T, Hirata Y, Ihara S, Suzuki N, Kinoshita H, Hayakawa Y (2017). Prevalence of pks-positive Escherichia coli in Japanese patients with or without colorectal cancer. Gut Pathog.

[13] Raisch J (2014). Colon cancer-associated B2 Escherichia coli colonize gut mucosa and promote cell proliferation. WJG.

[14] Buc E, Dubois D, Sauvanet P, Raisch J, Delmas J, Darfeuille-Michaud A (2013). High prevalence of mucosa-associated E. coli producing cyclomodulin and genotoxin in colon cancer. PloS One.

[15] Maddocks OD, Scanlon KM, Donnenberg MS (2013). An Escherichia coli effector protein promotes host mutation via depletion of DNA mismatch repair proteins. mBio.

[16] Cougnoux A, Dalmasso G, Martinez R, Buc E, Delmas J, Gibold L (2014). Bacterial genotoxin colibactin promotes colon tumour growth by inducing a senescence-associated secretory phenotype. Gut.

[17] Bandehpour M, Mirzarazi M, Hashemi A, Vahidi M, Taghavi A, Bashiri S (2021). Antibiotic resistance, phylogenetic group, and genotyping investigation in Escherichia coli strains of gut flora in patients with colorectal cancer in Iranian population. Biomed Biotechnol Res (BBRJ).

[18] Tae Jeong Oh IGK, Seon Young Park, Kug Chan Kim, Hye Won Shim (2002). NAD-dependent malate dehydrogenase protects against oxidative damage in Escherichia coli K-12 through the action of oxaloacetate. Environ Toxicol Pharmacol.

[19] Sreevalsan S, Safe S (2013). Reactive oxygen species and colorectal cancer. Curr Colorectal Cancer Rep.

[20] Zhang Boxi, Tornmalm Johan, Widengren Jerker, Vakifahmetoglu-Norberg Helin, Norberg Erik (2017). Characterization of the role of the malate dehydrogenases to lung tumor cell survival. J Cancer.

[21] Todisco Simona, Convertini Paolo, Iacobazzi Vito, Infantino Vittoria (2019). TCA cycle rewiring as emerging metabolic signature of hepatocellular carcinoma. Cancers.

[22] Zhuang Y, Xiang J, Bao W, Sun Y, Wang L, Tan M (2017). MDH2 stimulated by Estrogen-GPR30 pathway down-regulated PTEN expression promoting the proliferation and invasion of cells in endometrial cancer. Transl Oncol.

[23] Wu Hsueh-Hsia, Yi-Yuan Yang, Hsieh Wen-Shyang, Lee Chi-Hsin, Leu Sy-Jye C, Chen Mei-Ru (2009). OmpA is the critical component for escherichia coli invasionyinduced astrocyte activation. J Neuropathol Exp Neurol.

[24] Torres AG, Li Y, Tutt CB, Xin L, Eaves-Pyles T, Soong L (2006). Outer membrane protein a of escherichia coli O157:H7 stimulates dendritic cell activation. Infect Immun.

[25] Cierpicki T, Liang B, Tamm LK, Bushweller JH (2006). Increasing the accuracy of solution NMR structures of membrane proteins by application of residual dipolar couplings high-resolution structure of outer membrane protein A. J Am Chem Soc.

[26] Ashish Arora FA, Bushweller John H, Tamm Lukas K (2001). Structure of outer membrane protein A transmembrane domain by NMR spectroscopy. Nat Struct Mol Biol.

[27] Maria U, Johansson SA, Kaifeng HU, Walser Reto, Koebnik Ralf, Pervushin Konstantin (2007). A minimal transmembrane β-barrel platform protein studied by nuclear magnetic resonance. Biochemistry.

[28] AlexPautscha aGE (2000). High-resolution structure of the OmpA membrane domain. J Mol Biol.

[29] Schulz APaGE (1998). Structure of the outer membrane protein A transmembrane domain. Nat Struct Mol Biol.

[30] Zhao H, Sequeira RD, Galeva NA, Tang L (2011). The host outer membrane proteins OmpA and OmpC are associated with the Shigella phage Sf6 virion. Virology.

[31] Tan K, Deatherage Kaiser BL, Wu R, Cuff M, Fan Y, Bigelow L, Jedrzejczak RP, Adkins JN, Cort JR, Babnigg G, Joachimiak A (2017). Insights into PG-binding, conformational change, and dimerization of the OmpA Cterminal domains from Salmonella enterica serovar Typhimurium and Borrelia burgdorferi. Protein Sci..

[32] Alexander JL, Scott AJ, Pouncey AL, Marchesi J, Kinross J, Teare J (2018). Colorectal carcinogenesis: an archetype of gut microbiota-host interaction. Ecancermedicalscience.

[33] Eklof V, Lofgren-Burstrom A, Zingmark C, Edin S, Larsson P, Karling P (2017). Cancer-associated fecal microbial markers in colorectal cancer detection. Int J Cancer.

[34] Coleman OI, Nunes T (2016). Role of the microbiota in colorectal cancer: updates on microbial associations and therapeutic implications. BioResearch Open Access.

[35] Mirzarazi M, Rezatofighi SE, Pourmahdi M, Mohajeri MR (2015). Occurrence of genes encoding enterotoxins in uropathogenic Escherichia coli isolates. Braz J Microbiol.

[36] Mainil J (2013). Escherichia coli virulence factors. Vet Immunol Immunopathol.

[37] Zhang L, Yu J (2013). Role of apoptosis in colon cancer biology, therapy, and prevention. Curr Colorectal Cancer Rep.

[38] Bohlul E, Hasanlou F, Taromchi AH, Nadri S (2019). TRAIL-expressing recombinantLactococcus lactisinduces apoptosis in human colon adenocarcinomaSW480 andHCT116 cells. J Appl Microbiol.

[39] Vijayendran C, Burgemeister S, Friehs K, Niehaus K, Flaschel E (2007). 2DBase: 2D-PAGE database of Escherichia coli. Biochem Biophys Res Commun.

[40] Perez-Llarena FJ, Bou G (2016). Proteomics as a tool for studying bacterial virulence and antimicrobial resistance. Front Microbiol.

[41] Russo R, Valletta M, Rega C, Marasco R, Muscariello L, Pedone PV (2019). Reliable identification of lactic acid bacteria by targeted and untargeted high-resolution tandem mass spectrometry. Food Chem.

[42] Brewer LK, Jones JW, Blackwood CB, Barbier M, Oglesby-Sherrouse A, Kane MA (2020). Development and bioanalytical method validation of an LC-MS/MS assay for simultaneous quantitation of 2-alkyl-4(1H)-quinolones for application in bacterial cell culture and lung tissue. Anal Bioanal Chem.

[43] Khalid S, Bond PJ, Carpenter T, Sansom MSP (2008). OmpA: Gating and dynamics via molecular dynamics simulations. Biochim Biophys Acta Biomembr.

[44] Hagan EC, Mobley HLT (2007). Uropathogenic escherichia coli outer membrane antigens expressed during urinary tract infection. Infect Immun.

[45] Yilmaz ES, Aslantas O (2020). Phylogenetic group/subgroups distributions, virulence factors, and antimicrobial susceptibility of escherichia coli strains from urinary tract infections in Hatay. SBMT.

[46] Bakhshi M, Zandi H, Bafghi MF, Astani A, Ranjbar VR, Vakili M (2020). A survey for phylogenetic relationship; presence of virulence genes and antibiotic resistance patterns of avian pathogenic and uropathogenic Escherichia coli isolated from poultry and humans in Yazd. Iran Gene Reports.

[47] Khan S, Zaidi S, Alouffi AS, Hassan I, Imran A, Khan RA (2020). Computational proteome-wide study for the prediction of escherichia coli protein targeting in host cell organelles and their implication in development of colon cancer. ACS Omega.

[48] Abdelhai H, Comparative M (2016). Study of Rapid DNA extraction methods of pathogenic bacteria. Am J Biosci Bioeng.

[49] Mishra AK, Singh DD, Kumarsen G, Gupta G, Sharma N, Kumar N (2017). UspA gene based characterization of escherichia coli strains isolated from different disease conditions in goats. Int J Anim Res.

[50] Mirzarazi M, Rezatofighi SE, Pourmahdi M, Mohajeri MR (2013). Antibiotic resistance of isolated gram negative bacteria from urinary tract infections (UTIs) in Isfahan. Jundishapur J Microbiol.

[51] Jajarmi M, Ghanbarpour R, Sharifi H, Golchin M (2015). Distribution pattern of EcoR phylogenetic groups among shiga toxin-producing and enteropathogenic escherichia coli isolated from healthy goats. Int J Enteric Pathog.

[52] Clermont O, Bonacorsi S, Bingen E (2000). Rapid and simple determination of the escherichia coli phylogenetic group. Appl Environ Microbiol.

[53] Sadeghi M, Bandehpour M, Yarian F, Yassaee V, Torbati E, Kazemi B (2014). Cloning and expression of influenza h1n1 ns1 protein in escherichia coli BL21. Iran J Biotechnol.

[54] Yazdanfar M, Bandehpour M, Yarian F, Koochaki A, Parivar K, Kazemi B (2010). Cloning and expression of human vascular endothelial growth factor gene and inhibition of its expression by antisense in prokaryotic system. Daru.

[55] Wiese S, Reidegeld KA, Meyer HE, Warscheid B (2007). Protein labeling by iTRAQ: a new tool for quantitative mass spectrometry in proteome research. Proteomics.

[56] Ramos S, Silva N, Hébraud M, Santos HM, Nunes-Miranda JD, Pinto L (2016). Proteomics for drug resistance on the food chain? multidrug-resistant escherichia coli proteomes from slaughtered pigs. OMICS.

[57] Curreem SOT, Watt RM, Lau SKP, Woo PCY (2012). Two-dimensional gel electrophoresis in bacterial proteomics. Protein Cell.

[58] Wang H, Cheng X, Zhang L, Xu S, Zhang Q, Lu R (2019). A surface-layer protein from Lactobacillus acidophilus NCFM induces autophagic death in HCT116 cells requiring ROS-mediated modulation of mTOR and JNK signaling pathways. Food Funct.

[59] Bringans S, Eriksen S, Kendrick T, Gopalakrishnakone P, Livk A, Lock R (2008). Proteomic analysis of the venom ofHeterometrus longimanus (Asian black scorpion). Proteomics.

[60] Magalhaes S, Aroso M, Roxo I, Ferreira S, Cerveira F, Ramalheira E (2017). Proteomic profile of susceptible and multidrug-resistant clinical isolates of Escherichia coli and Klebsiella pneumoniae using label-free and immunoproteomic strategies. Res Microbiol.

[61] Pak JN, Jung JH, Park JE, Hwang J, Lee HJ, Shim BS, Kim SH (2020). p53 dependentLGR5 inhibition and caspase 3 activation are critically involved in apoptotic effect of compound K and its combination therapy potential in HCT116 cells. Phytother Res..

